# The analysis of risk factors for diabetic nephropathy progression and the construction of a prognostic database for chronic kidney diseases

**DOI:** 10.1186/s12967-019-2016-y

**Published:** 2019-08-13

**Authors:** Gang Wang, Jian Ouyang, Shen Li, Hui Wang, Baofeng Lian, Zhihong Liu, Lu Xie

**Affiliations:** 1 Division of Nephrology, Jinling Hospital, Southern Medical University, Nanjing, 210016 China; 2 National Clinical Research Center of Kidney Diseases, Jinling Hospital, Nanjing University School of Medicine, Nanjing, 210016 China; 3 Shanghai Center for Bioinformation Technology, Shanghai Academy of Science and Technology, Shanghai, 201203 China

**Keywords:** Diabetic nephropathy, Progression, Risk factor, Prognostic marker, Bioinformatics analysis, Database

## Abstract

**Background:**

Diabetic nephropathy (DN) affects about 40% of diabetes mellitus (DM) patients and is the leading cause of chronic kidney disease (CKD) and end-stage renal disease (ESRD) all over the world, especially in high- and middle-income countries. Most DN has been present for years before it is diagnosed. Currently, the treatment of DN is mainly to prevent or delay disease progression. Although many important molecules have been discovered in hypothesis-driven research over the past two decades, advances in DN management and new drug development have been very limited. Moreover, current animal/cell models could not replicate all the features of human DN, while the development of Epigenetics further demonstrates the complexity of the mechanism of DN progression. To capture the key pathways and molecules that actually affect DN progression from numerous published studies, we collected and analyzed human DN prognostic markers (independent risk factors for DN progression).

**Methods:**

One hundred and fifty-one DN prognostic markers were collected manually by reading 2365 papers published between 01/01/2002 and 12/15/2018. One hundred and fifteen prognostic markers of other four common CKDs were also collected. GO and KEGG enrichment analysis was done using g:Profiler, and a relationship network was built based on the KEGG database. Tissue origin distribution was derived mainly from The Human Protein Atlas (HPA), and a database of these prognostic markers was constructed using PHP Version 5.5.15 and HTML5.

**Results:**

Several pathways were significantly enriched corresponding to different end point events. It is shown that the TNF signaling pathway plays a role through the process of DN progression and adipocytokine signaling pathway is uniquely enriched in ESRD. Molecules, such as TNF, IL6, SOD2, etc. are very important for DN progression, among which, it seems that “AGER” plays a pivotal role in the mechanism. A database, dbPKD, was constructed containing all the collected prognostic markers.

**Conclusions:**

This study developed a database for all prognostic markers of five common CKDs, offering some bioinformatics analyses of DN prognostic markers, and providing useful insights towards understanding the fundamental mechanism of human DN progression and for identifying new therapeutic targets.

**Electronic supplementary material:**

The online version of this article (10.1186/s12967-019-2016-y) contains supplementary material, which is available to authorized users.

## Background

DN, also known as “diabetic kidney disease (DKD)”, is one of the most important diabetic microvascular complications, affecting 30–45% patients with either type 1 DM (T1DM) or type 2 DM (T2DM), with a peak incidence in the 10–20 years duration of DM [1–3]. DN, pathologically, is often characterized by glomerular basement membrane (GBM) thickening, glomerular mesangial matrix expansion, and formation of glomerular nodular sclerosis in its advanced stages [4], and clinically, is usually defined by proteinuria occurrence or declined renal function, e.g. reduced glomerular filtration rate (GFR) [1, 5]. DN patients exhibiting modest or no albuminuria may progress to ESRD [6, 7]. DN is the leading cause of CKD and ESRD in high-income countries and likely worldwide [8–11], and also a single strong predictor of mortality in patients with DM [12]. Even worse, the absolute number of DN patients continues to increase and the incidence of ESRD from DN keeps expanding [13], consistent with the global DM pandemic [9, 14].

Currently, tight glucose control and strict blood pressure control (especially with medications that inhibit the renin-angiotensin system) remain the mainstay of management for DN. Although some progress has been made in reducing diabetes-related mortality and delaying the development of kidney disease from DM, the percentage of DN patients who progress to ESRD has not substantially declined [5]. Disappointingly, there has been an impasse in the development of new drugs for DN, with no success in Phase 3 clinical trials [15]. One reason is the lack of accurate understanding of the underlying pathophysiological mechanisms of human DN development and progression. Targeting single molecules and/or pathways that were important in DN development and progression based on hypothesis-driven research has not yielded significant advances in DN treatment in the past two decades. On one hand, mechanisms underlying DN development and progression are complicated with many interacting molecules and a number of crosstalk pathways. On the other hand, current animal and cell culture models mainly replicate the early stage of and/or recapitulate certain features of human DN, failing to reproduce the whole process of DN development and progression [16]. In addition, patients who strictly complied with treatment recommendations can still develop overt DN whereas patients with similar or poor compliance may not. Likewise, not all DM patients with microalbuminuria progress to macroalbuminuria or ESRD (some patients even revert and the microalbuminuria disappears). Therefore, more broad-based approaches including systems biology and multiple omics are being applied to understanding DN pathological mechanisms today [17–19].

Regarding this situation, we collected all DN prognostic markers (risk factors for DN progression) from both routine and high-throughput research based on human samples in the past two decades and performed additional bioinformatics analyses, hoping to offer some insights into the mechanism of DN progression, which might help DN research and the discovery of new therapeutic targets for DN.

We constructed a database dbPKD [20], for prognostic markers of DN, as well as other CKDs including IgA nephropathy (IgAN), idiopathic membranous nephropathy (IMN), primary focal segmental glomerulosclerosis (pFSGS) and Lupus nephritis (LN). There have been no previously focused databases for risk factors of kidney diseases. dbPKD may provide a resource for searching reported prognostic factors for common CKDs.

## Methods

### Data collection

All DN prognostic markers (risk factors for DN progression) were collected by screening through related literature. We searched the PubMed database using 32 keywords, e.g. “DN”, “DKD”, “diabetic kidney disease”, “diabetic nephropathy”, “ESRD”, “marker”, etc. (Additional file 1: Table S1). Totally, 2365 papers published between 01/01/2002 and 12/15/2018 were collected, including both routine research and high-throughput research. Reviews and non-English literature were excluded first. Initial screening of literature was based on title and abstract. Four hundred and three papers were retained for further filtration. Their contents were checked for information in detail. Filtrations were carried out according to rules: (1) the research subjects must be human, that is, samples used for the prognosis study must be derived from humans; (2) the disease studied must be DN, or a synonym of its definition, such as DKD; (3) markers must be potentially prognostic, which means that these markers should be potential risk/protective factors of GFR decline, doubling of serum creatinine, CKD progression, ESRD or even death closely related to kidney damage. In addition, markers used to predict significant albuminuria/proteinuria progression in DN patients were also included; (4) only markers that were rigorously verified to be independent risk factors for DN progression in multivariate analysis were finally collected, and several markers with only univariate analysis results in current prognostic studies were also collected; (5) markers of multiple omic-levels were collected, including genes (involving mRNA, SNP, CNV, etc.), proteins, microRNAs, and mixed clinical indicators (referring to all the prognostic markers that are not genes, proteins, or microRNAs).

Besides DN prognostic markers, we also collected prognostic markers of other four CKDs (IgAN, IMN, pFSGS and LN). The collection guidelines were basically the same as that for DN data. However, there were several different points as follows: (1) the key words are shown in Additional file 1: Table S2; (2) papers published between 01/01/2002 and 01/01/2018 were filtered for IgAN, IMN, pFSGS and LN; (3) markers must be prognostic for GFR decline, doubling of serum creatinine, CKD progression, ESRD or even death closely related to kidney damage, but not necessarily prognostic for albuminuria/proteinuria progression. The workflow for data processing is shown in Additional file 1: Figure S1.

### Functional enrichment analysis of DN prognostic molecules

We performed GO and KEGG enrichment analysis for DN prognostic molecules using a test based on the hypergeometric distribution, with false discovery rate (FDR) < 0.05 being considered significant. All this work was done using the g:Profiler platform [21].

### Network analysis

In order to analyze the connectivity and co-regulation among the DN prognostic molecules, we constructed a network according to the main enriched pathways in DN progression based on KEGG [22] using Edraw Version 9.3.0.0 [23]. We also manually constructed a signal-transduction diagram by extracting the regulatory relationship from the enriched signal transduction pathways to illustrate the speculated role of prognostic molecules in DN progression more clearly.

### Tissue origin distribution

To establish the expression and location of prognostic molecules in normal kidney tissues, we searched all prognostic genes and proteins in the HPA [24]. First, we downloaded the mRNA and protein data for all genes in different human systems/tissues from the HPA, and then screened out kidney tissue (e.g. glomeruli, tubules, etc.) related data. Finally, we obtained the expression levels and location data of prognostic genes and proteins in kidney tissues by molecule ID mapping.

### Database construction

To avoid duplication and to unify the naming of markers across different studies, genes were mapped to Entrez Gene IDs, and proteins were mapped to UniProt IDs. Mixed clinical indicators were given unified names if these are widely used. Sample sources were categorized into renal tissue, urine and blood (including serum and plasma), and the prognostic effects were mainly divided into “better” and “worse”. All the collected data were incorporated into the database after collation and normalization, and each entry included five types of information: reference, research parameters, marker annotation, prognostic effect(s) and the supportive public data.

The web interface for dbPKD was developed using PHP Version 5.5.15 and HTML5. JavaScript and jQuery were also used to enable dynamic web services. The database was implemented in MySQL Server 5.5.48 and deployed in Apache web server running on the CentOS 6.5 system. Data analyses were mainly developed using R script.

The web interface mainly provides four types of application service: Browse, Search, Analysis and Download.

## Results

### Data statistics

In total, for DN progression, without distinguishing specimen sources, 46 genes, 42 proteins, 3 microRNAs, and 60 mixed clinical indicators were manually collected from 115 qualified papers published between 01/01/2002 and 12/15/2018. Most DN prognosis studies were multi-centered, and were mainly located in Europe, North America and East Asia. According to the primary DM subtypes, the DN study population could be divided into three subgroups: T1DN, T2DN and undefined DN. Specially, the undefined DN subgroup indicates that the study population did not include an independent, well-defined T1DN (secondary to T1DM) cohort or T2DN (secondary to T2DM) cohort. The prognostic markers could also be divided into three groups based on the DN population (Additional file 1: Figure S2). Only one gene (ACE) and six proteins (ADIPOQ, CST3, TNNT2, TNFRSF1A, FABP1, HBB) were verified as potentially prognostic in both T1DN and T2DN (Table 1).

**Table 1:** Genes, proteins and microRNAs verified in T1DN and T2DN, respectively

	T1DN	Common	T2DN
Gene	AGER, ATP5MC3, BDKRB2, CASP3, CAT, CCR5, CNDP1, COX5A, CTGF, CYP11B2, ENPP1, FLT4, GPX1, HPSE, LIPC, NPHS1, NPPA, PARP1, SLC2A1, SOD1, SOD2, TGFBR2, TRPC6, UQCRC1, CDH13, CYBA	ACE	ADIPOQ, AKR1B1, APOE, CCL2, CETP, GSTT1, IL10, ITGA2, LTA, NOS3, PON1, PON2, PRKCB, SLC12A3, TKT, FN3K, EP300, HP
microRNA			miR-126, miR-196a, miR-9
Protein	CRP, CTGF, MBL2, TNFRSF11B, UMOD	ADIPOQ, CST3, TNNT2, TNFRSF1A, FABP1, HBB	CLU, COL18A1, CP, FGF21, HP, ICAM1, IL6, TNFRSF1B, CD59, CFHR2, C4A, MCAM, LGALS3, AVP, NPPB, RBP4, SAA1, TNF, VCAM1, VWF, C8A, AOC3, FGF23, SERPINF1, VEGFA, ALB, CCL2

**Fig. 1:**
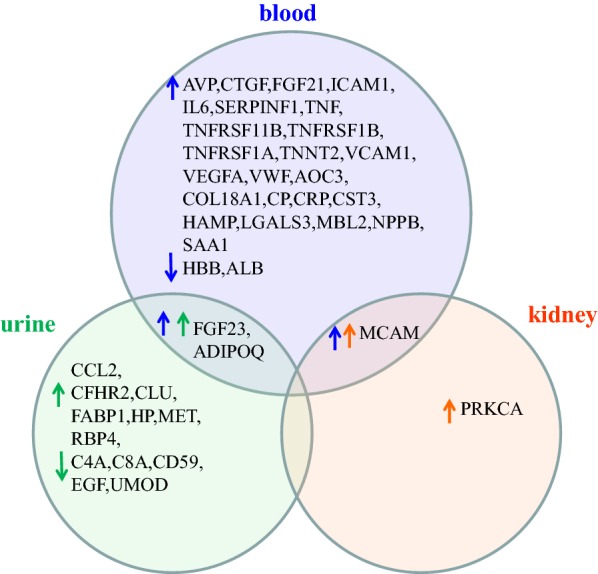
Specimen sources of prognostic proteins and their observed increase/decrease associated with worse prognosis of DN. Blue arrow represents protein change in blood, green arrow is for urine specimen, and orange arrow for kidney tissue

Without distinguishing amongst DN subtypes, almost all prognostic genes were verified using human blood specimens, while prognostic proteins were verified mainly based on blood and urine specimens (Additional file 1: Figure S3). Specifically in these DN prognosis studies, two proteins, FGF23 and ADIPOQ, were increased in both blood and urine to predict “worse” prognosis of DN, while one other protein, MCAM, was observed to be “positive” in kidney tissue and “level-increased” in blood (Fig. 1). Additionally, four molecules, ADIPOQ, CCL2, CTGF and HP, were verified as potentially prognostic for DN progression in both gene and protein levels (Additional file 1: Figure S4).

### Molecules involved in DN progression and the functional analyses

**Fig. 2:**
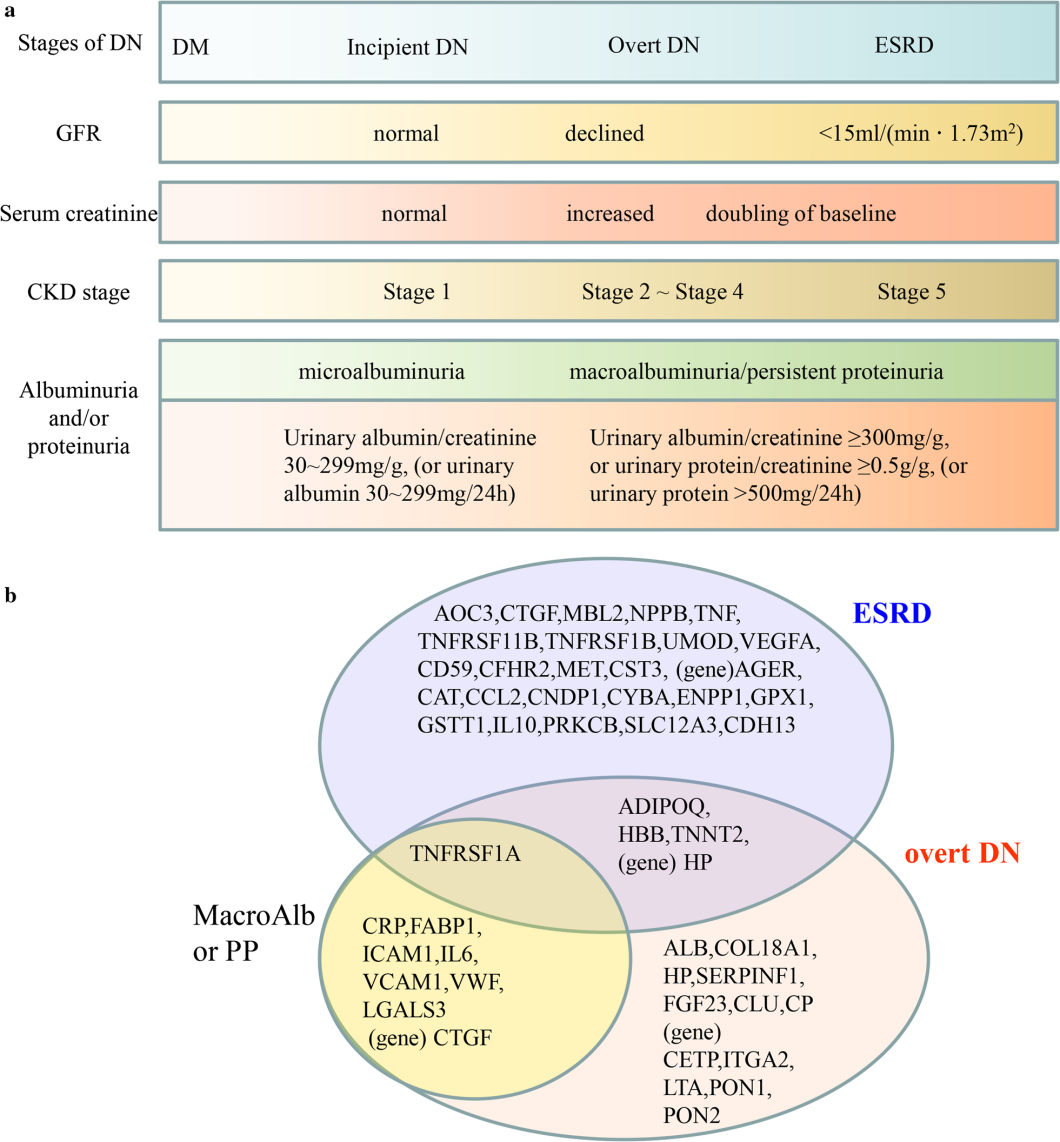
Grouping based on the end point events and corresponding clinical parameters. **a** End point events and corresponding clinical parameters. **b** Grouping of DN prognostic genes and proteins according to the end point events involved in different studies

Based on the DN classification [25] in 2014 and a preliminary analysis of all defined end point events in the collected papers (Fig. 2a), the prognostic genes and proteins could be divided into several groups. Among them, two groups were of particular interest: the ESRD group, and the overt DN group (referring to a group of molecules that were prognostic for GFR decline not reaching ESRD). Specially, molecules prognostic for only albuminuria/proteinuria progression were clustered as MacroAlb/PP group (macroalbuminuria/persistent proteinuria as the end point event). Interestingly, prognostic molecules in the MacroAlb/PP group were basically included in the overt DN group (Fig. 2b).

**Fig. 3:**
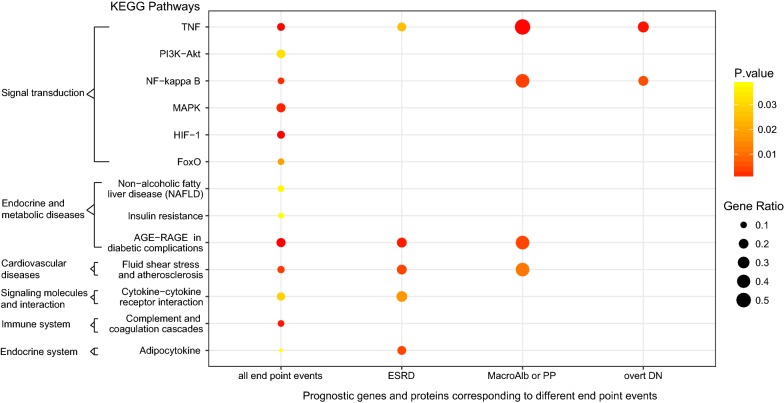
KEGG enrichment analysis of DN prognostic genes and proteins corresponding to different end point events. TNF signaling pathway, PI3K-Akt signaling pathway, NF-kappa B signaling pathway, MAPK signaling pathway, HIF-1 signaling pathway and FoxO signaling pathway all belong to “Signal transduction” pathways

We performed GO and KEGG enrichment analysis. In total, six “Signal transduction” pathways, three “Endocrine and metabolic diseases” pathways, one “Immune system” pathway, one “Endocrine system” pathway, and also one “Cardiovascular diseases” pathway were significantly enriched (p < 0.05) (Fig. 3, Additional file 1: Figure S5). Interestingly, as shown in Fig. 3, TNF signaling pathway was enriched in MacroAlb/PP group, overt DN group and ESRD group, which suggested that it play an important role through DN progression from the occurrence of microalbuminuria to ESRD. Similarly, NF-kappa B signaling pathway enriched in MacroAlb/PP group and overt DN group but not ESRD group might be primarily involved in the early stages of DN progression. Moreover, almost all of the pathways enriched in MacroAlb/PP group were enriched in ESRD group, implying that there should be a close relationship between the mechanism of proteinuria progression and that of ESRD development, which also, from the perspective of bioinformatics, confirmed that proteinuria was a risk factor for adverse renal outcome [26, 27]. Furthermore, prognostic genes and proteins of all the GFR-decline related end point events (including GFR decline, serum creatinine rise, ESRD, start of replacement therapy, and a more serious CKD stage/DN stage) were significantly enriched in HIF-1 signaling pathway, MAPK signaling pathway, TNF signaling pathway, AGE-RAGE signaling pathway in diabetic complications and “Fluid shear stress and atherosclerosis” pathway (not shown), indicating that the Cardiovascular diseases pathway “Fluid shear stress and atherosclerosis” might be activated in DN progression, this, to a certain extent, explained the risk of cardiovascular death in DN patients [28]. In addition, referring to the adipocytokine signaling pathway enriched in ESRD group, there have been several adipocytokines reported to participate in DN development and progression in recent years. One of them was adiponectin (ADIPOQ), besides being verified as a prognostic molecule in DN prognosis studies [29–31], it was observed increased in the serum of DN patients, protected the kidney by reducing inflammatory response and ameliorating glomerular hypertrophy and albuminuria, as an anti-inflammatory adipokine and insulin sensitizer mainly secreted by adipocytes [32]. There were also some other adipocytokines reported, such as visfatin and apelin. Visfatin, or pre-B cell colony-enhancing factor, is synthesized in adipocytes, had an important paracrine role in the development of DN through inducing tyrosine phosphorylation of the insulin receptor, activating downstream insulin signaling pathways and increasing the levels of TGF beta1, PAI-1, type I collagen, and MCP-1 (CCL2) [33]. Apelin contributed to DN progression by inhibiting autophagy in podocytes [34].

Although there are many biological processes (BPs) involved in DN progression, we only focused on the top 15 BPs significantly enriched for all the DN prognostic genes and proteins (Additional file 1: Figure S5). It is noted that the risk molecules in MacroAlb/PP group were mainly enriched in 5 of the top 15 BPs: response to stress, inflammatory response, response to oxygen-containing compound, response to lipid, and regulation of cell death, which might indicate that inflammation, oxidative stress, hemodynamics abnormality, and lipid metabolism disorder had been damaging the kidney function since the very early DN stage with albuminuria occurrence.

### Risk molecules for different DN stages based on different end point events

According to the three clusters of DN prognostic molecules, based on different end point events (Fig. 2b), we could observe different risk molecules for specific DN stages. There were very few overlapping risk molecules between the ESRD group and the overt DN group, which indicated that there might be different key molecules promoting DN progression at different DN stages. For example, CTGF was verified as a risk gene for albuminuria progression [35] and a risk protein for progressing to ESRD [36]. Studies using animal/cell models show that CTGF could be induced by high glucose through the mediation of TGF-β, and its upregulation could promote mesangial matrix accumulation, progressive glomerulosclerosis and tubulointerstitial fibrosis [37, 38]. In podocytes, its overexpression could damage podocytes and exacerbate proteinuria and mesangial expansion [39]. Considering all the above observations, it is speculated that CTGF should exert a very weak or no effect on the promotion of DN progression in the early albuminuria stage of DN, although it was a risk gene for albuminuria progression, while in the middle and late DN stages, CTGF should act as a key molecule promoting the development of ESRD and play an very important role in DN progression.

### Role of DN prognostic markers in the mechanism of DN progression

**Fig. 4:**
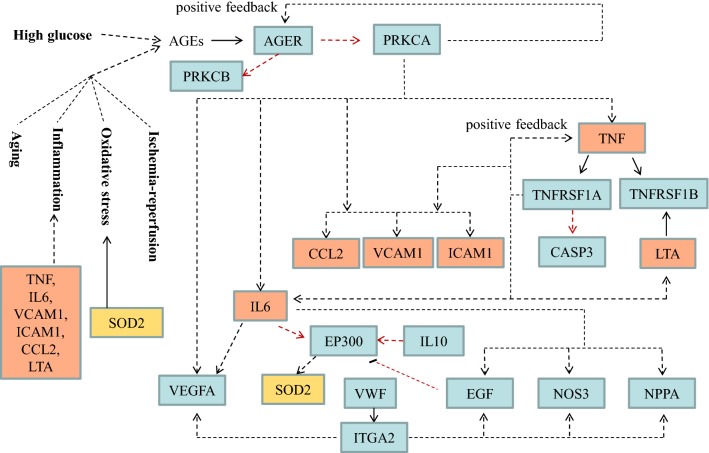
Overview of regulatory relationships among DN prognostic molecules in enriched signal transduction pathways. Solid line represents molecular interaction or relation. Dotted line represents indirect link, state change or unknown reaction. Red line represents link in the cytoplasm. Molecule in the rectangle represents gene product, mostly protein but including RNA

We constructed a network according to the aforementioned KEGG pathways (Fig. 3) to show the connections and regulation among DN prognostic molecules (Additional file 1: Figure S6). To illustrate the role of DN prognostic molecules in the mechanism of DN progression more clearly, we also drew a signal-transduction diagram by extracting the regulatory relationship from the enriched signal transduction pathways (Fig. 4). For the integrity of the regulation loop, AGE-RAGE signaling pathway in diabetic complications is also included in the diagram. As shown in Fig. 4, it seems that AGER, interacting with AGEs initially produced by high blood glucose, is an important molecule in DN development. Also, AGER plays a pivotal role in the subsequent DN progression mechanism, just like the “switch” in the regulation loop. Accumulation/activation of TNF, TNFRSF1A and IL6 could still promote DN progression even without the existence of high blood glucose. Actually, the role of some of the DN prognostic molecules in the mechanism of DN development and progression and their regulatory relationship have been studied in the past two decades using animal and cell culture models (Additional file 1: Figure S7) [40–51]. For example, TNF could cause cholesterol-dependent podocyte apoptosis and albuminuria, which was mediated by nuclear factor of activated T cells 1 (NFATc1) [52]. Blockade of macrophage-derived TNF could protect kidney and reduce albuminuria and plasma creatinine in a diabetic mouse model [53]. CRP could be induced by high blood glucose and significantly upregulate TNF, CCL2 and CTGF via CD32a/64 in vitro. CRP transgenic mice developed more severe DN with increased albuminuria and enhanced renal inflammation compared to wild-type mice [41]. In addition, PEDF (SERPINF1) could inhibit tubular cell injury by suppressing RAGE (AGER) expression in streptozotocin-induced diabetic rats [45], while EGF could prevent podocyte apoptosis induced by high glucose [54].

### Protein expression and location of DN prognostic genes and proteins

**Fig. 5:**
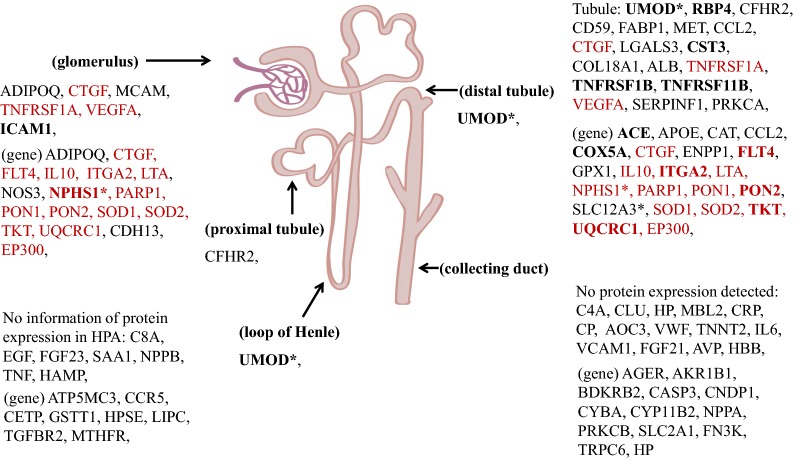
Protein expression and location of DN prognostic molecules in renal tissues using the HPA [24]. The asterisk (*) denotes specific protein expression in kidney. Bold indicates high protein expression, and proteins expressed in both glomeruli and tubules are in red

In order to explore the law/characteristic of the spatial distribution in DN prognostic molecules, we located all DN prognostic genes and proteins in renal tissues using the HPA [24] (Fig. 5). As shown in Fig. 5, most of the DN prognostic molecules are expressed in normal kidneys and could be found in the HPA query. Some of them have high protein expression in normal kidneys, for example, ICAM1 and NPHS1 are high expressed in normal glomeruli, while UMOD, RBP4, CST3, TNFRSF1B, TNFRSF11B, ACE, COX5A, ITGA2, PON2, TKT, UQCRC1 are high expressed in tubules. And several molecules are expressed in normal kidneys but not in other human normal tissues: NPHS1, UMOD, and SLC12A3. Moreover, most of the prognostic genes expressed in normal kidneys could be found in both glomeruli and tubules. Interestingly, almost all of the prognostic proteins verified only through urine specimens are expressed in normal renal tubules, except C4A, CLU and HP (with C8A and EGF unknown), which suggests that DN progression might be closely related to the dysregulation of protein expression that originally existed in normal kidneys.

### dbPKD: database for prognostic markers of kidney diseases [20]

In total, 69 genes, 72 proteins, 4 microRNAs, and 92 mixed clinical indicators were extracted from 243 qualified papers, without distinguishing specimen sources. And 46 genes, 42 proteins, 3 microRNAs, and 60 mixed clinical indicators were extracted from 115 qualified papers for DN progression. In addition, 30 genes, 43 proteins, 1 microRNA, and 41 mixed clinical indicators were extracted from 128 qualified papers for IgAN, IMN, pFSGS and LN.

**Fig. 6:**
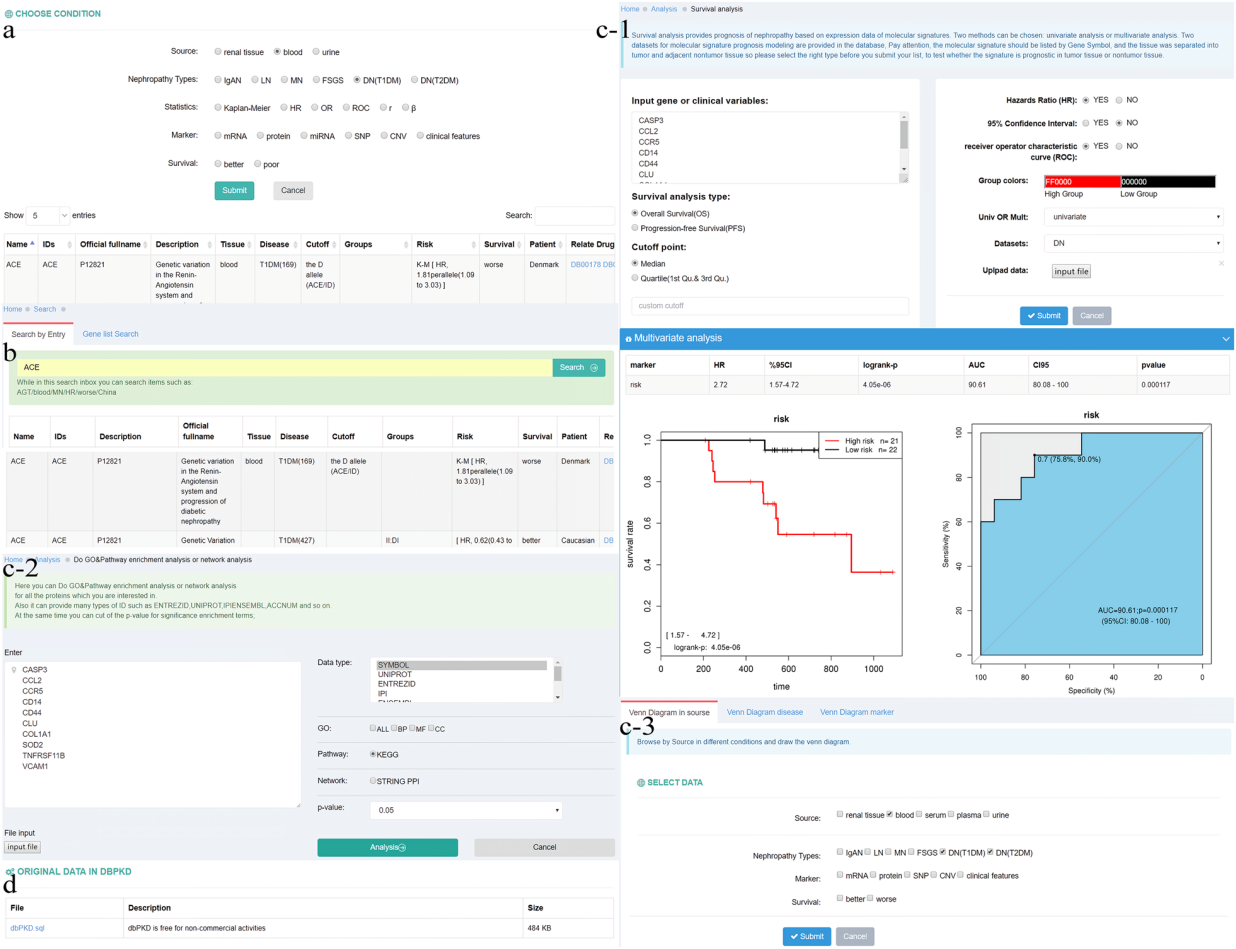
Web interfaces of the dbPKD. **a** The browse interface of dbPKD for prognostic markers in blood. **b** The search interface for a gene symbol. **c** The analysis interface which includes three modules: survival analysis, enrichment analysis and Venn analysis. **d** The download page of dbPKD with url and description

The browse interface provides data exploration to users across several features, such as specimen sources, marker types and prognosis effects etc. (Fig. 6a). Users can also search for one marker or a group of possible prognostic genes in the Search interface (Fig. 6b). Analysis interface provides users with three types of analysis service: survival analysis, enrichment analysis and Venn diagram analysis. In the “survival analysis” module, we pre-uploaded a 40-sample DN dataset obtained from National Clinical Research Center of Kidney Diseases (Nanjing) to provide a functional demonstration. Users can perform this analysis by 4 steps: (1) input a gene list, (2) select a dataset from the existing datasets or upload a user’s own dataset in the dataset panel, (3) choose other given conditions, and (4) click the “Submit”. Analysis results will be shown when the calculation is completed, including univariate and multivariate analysis tables, Kaplan–Meier survival curves, and prognosis models. In the “enrichment analysis” module, we provide GO and KEGG enrichment analysis as well as the protein–protein interaction (PPI) network analysis. Venn diagram analysis focuses on screening for common or specific markers in PKD research. The analysis can be performed mainly by one of the three conditions below: “Venn Diagram in source”, “Venn Diagram disease” and “Venn Diagram marker” (Fig. 6c). Finally, users can download data in Download interface (Fig. 6d). And dbPKD is free for non-commercial activities.

## Discussion

Theoretically, proper genetic intervention to DM patient might prevent DN from happening. However, resolving the genetics of DN remains complex with little progress. In the past decades, only a few molecules were identified as DN genetic factors through genome-wide association studies (GWAS), such as ACE, AKR1B1, APOE, PPARG, etc. [55]. Some of them were also verified as DN prognostic genes, which could be called “high-risk genes for DN development and progression” (Additional file 1: Figure S8). At present, in addition to strict management of diabetic patients, there seems to be no precautions for DN development. The main therapeutic strategy for DN patients is to inhibit or retard the disease progression. The prognostic markers collected here were all verified as risk factors for DN progression in DN prognosis studies. They were all directly related to the end point events of DN patients regardless of the complex interactions among molecules and Epigenetics. Hence, they might reflect the most real “key molecules” in DN progression and serve for finding new therapeutic targets. Analyzing these prognostic markers might offer some insights in understanding the mechanism of DN progression.

MicroRNAs are small non-coding RNA molecules that usually function in RNA silencing and post-transcriptional regulation by affecting their target mRNAs. Here we only collected three microRNAs that were verified as risk factors of DN progression. Interestingly, their target molecules included more DN prognostic genes and proteins [56] (Additional file 1: Figure S9), indicating that microRNAs should play an important role in DN progression. In some other related works, we confirmed the clinical application value of miR-196a for several types of kidney diseases [57, 58]. The regulation details between microRNAs and their targets as well as the possible associations among these three microRNAs need further research, which might help to understand the mechanism of DN progression. In addition, there were also some clinical indicators (including metabolites, biochemical indicators, pathological parameters, etc.) that could be used as DN prognostic markers. In fact, serum creatinine has been widely reported and clinically used as an important parameter in assessing and monitoring renal functions of kidney diseases for decades [59, 60]. Vitamin D has been discussed to be a treatment option in DN for many years [61, 62]. Both of these suggest that DN prognostic markers have potential important applications in the clinical diagnosis and treatment of DN.

Although we attempted to collect all the DN prognostic markers and analyze them as accurately as possible, there were still some limitations in our study. First, due to the limited prognosis studies, the number of DN prognostic molecules collected was small. Second, because of the fuzzy definitions of end point events, it was difficult to judge the accurate DN stages for which some prognostic markers were used. This also hindered subsequent further analysis. Lastly, specimen sources of risk factors for DN progression were variable, including urine, blood and kidney tissue, which posed difficulties for further mechanistic studies of DN progression.

The work on prognostic markers will be continued and the data is scheduled to be updated every 2 years. In the meantime, we will keep trying to improve the efficiency of data extraction by adopting some machine learning methods and endeavor to optimize the workflows. In addition, other types of related data, such as data from single cell sequencing studies, may also be collected in the subsequent work for further analysis. We hope that more prognostic markers of kidney diseases and valuable insights could be provided to clinicians and researchers.

## Conclusions

In conclusion, we collected human DN prognostic markers that were verified as independent risk factors of DN progression mostly through multivariate analysis in the past two decades and constructed a database. To our knowledge, this is the first systematic summary of DN prognostic markers. Bypassing the complex epigenetics and avoiding the shortcomings that animal/cell models could not replicate all the features of human DN, these prognostic molecules were directly related to human DN prognosis and were the most authentic key molecules in human DN progression. Also, we demonstrated the connections and regulation among these molecules and emphasized some related GO terms and KEGG pathways by bioinformatics analysis. The in-depth study of these molecules and related pathways will help to further understand the mechanism of human DN progression, discover new therapeutic targets and explore new DN drugs. In addition, some prognostic markers (mixed clinical indicators) might contribute to the improvement of the managements of DN patients. In the future, we will expand the data content and improve the functional modules for dbPKD, and strive to provide some more valuable insights for the research and treatment of related kidney diseases by adopting more and better analytical methods.

## Additional file

**Additional file 1: Table S1.** Key words for search of prognosis literature on DN in Pubmed. **Table S2.** Key words for search of prognosis literature on IgAN, IMN, pFSGS and LN in Pubmed. **Figure S1.** The workflow for data processing. **Figure S2.** Prognostic markers in DN subtypes. **Figure S3.** Specimen sources of DN prognostic markers. **Figure S4.** Prognostic genes and proteins of DN (Risk genes and proteins for DN progression). **Figure S5.** GO enrichment analysis of DN prognostic genes and proteins corresponding to different end point events. **Figure S6.** The distribution of DN prognostic molecules in the enriched KEGG pathways. **Figure S7.** Role and regulatory relationship of DN prognostic molecules based on animal/cell culture models. **Figure S8.** High-risk genes for DN development and progression. **Figure S9.** Interactions between DN prognostic microRNAs and proteins, genes.

## Data Availability

The datasets used and/or analyzed during the current study are available from the dbPKD or from the corresponding authors on reasonable request.

## Abbreviations

DN: diabetic nephropathy
DM: diabetes mellitus
CKD: chronic kidney disease
ESRD: end-stage renal disease
HPA: The Human Protein Atlas
DKD: diabetic kidney disease
T1DM: type 1 DM
T2DM: type 2 DM
GBM: glomerular basement membrane
GFR: glomerular filtration rate
IgAN: IgA nephropathy
IMN: idiopathic membranous nephropathy
pFSGS: primary focal segmental glomerulosclerosis
LN: Lupus nephritis
FDR: false discovery rate
ADIPOQ: adiponectin
BP: biological process
NFATc1: nuclear factor of activated T cells 1
PPI: protein–protein interaction
GWAS: genome-wide association study
