# Supplementary figures and images for: The analysis of risk factors for diabetic nephropathy progression and the construction of a prognostic database for chronic kidney diseases
Source: J Transl Med. 2019 Aug 13;17:264. doi: 10.1186/s12967-019-2016-y (PMC6693179; doi:10.1186/s12967-019-2016-y)

# GO Terms: Biological processes (BPs)

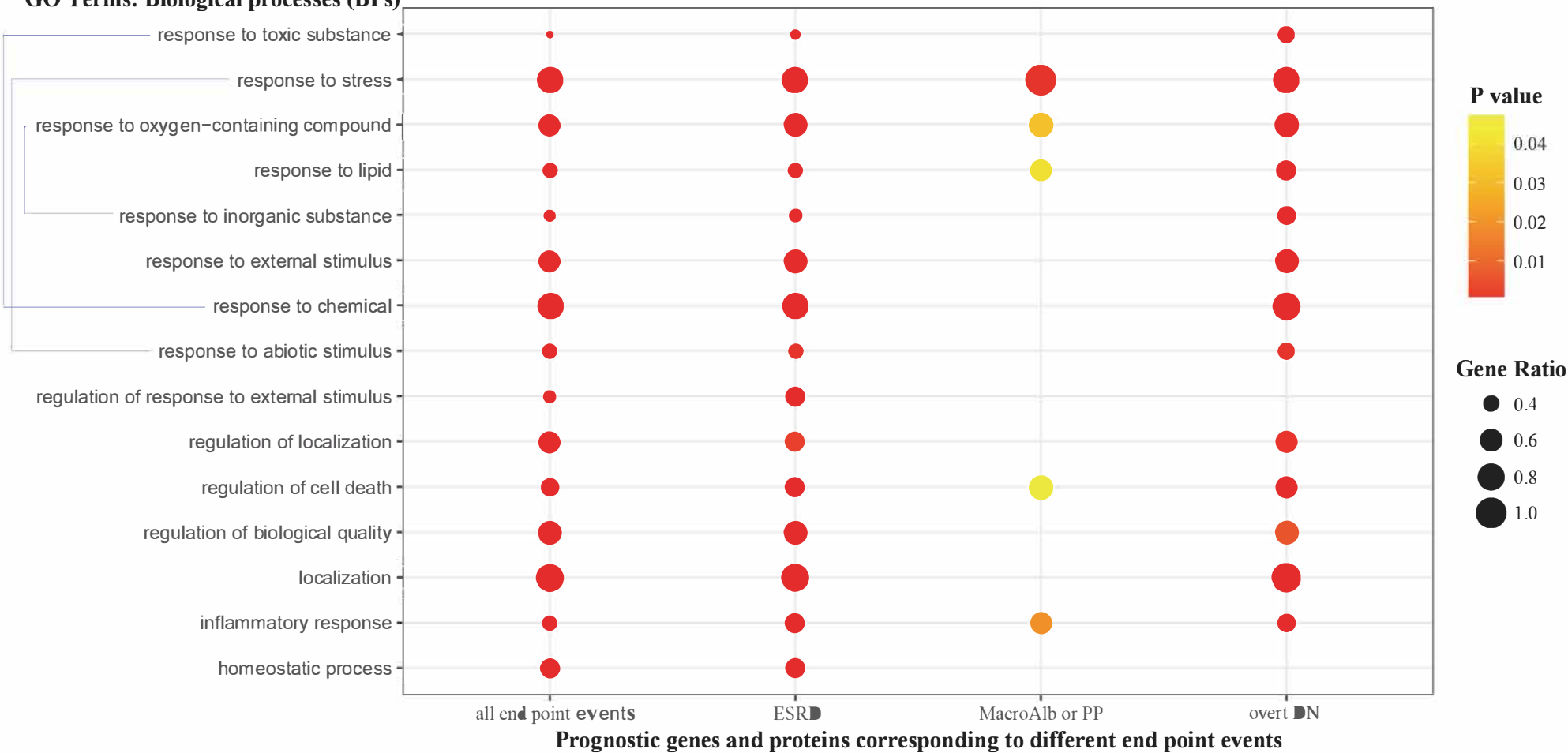

Supplement: Supplementary file 1 — Additional file 1: Table S1. Key words for search of prognosis literature on DN in Pubmed. Table S2. Key words for search of prognosis literature on IgAN, IMN, pFSGS and LN in Pubmed. Figure S1. The workflow for data processing. Figure S2. Prognostic markers in DN subtypes. Figure S3. Specimen sources of DN prognostic markers. Figure S4. Prognostic genes and proteins of DN (Risk genes and proteins for DN progression). Figure S5. GO enrichment analysis of DN prognostic genes and proteins corresponding to different end point events. Figure S6. The distribution of DN prognostic molecules in the enriched KEGG pathways. Figure S7. Role and regulatory relationship of DN prognostic molecules based on animal/cell culture models. Figure S8. High-risk genes for DN development and progression. Figure S9. Interactions between DN prognostic microRNAs and proteins, genes. [file 12967_2019_2016_MOESM1_ESM.zip › Additional file 1 Figure S5.pdf]

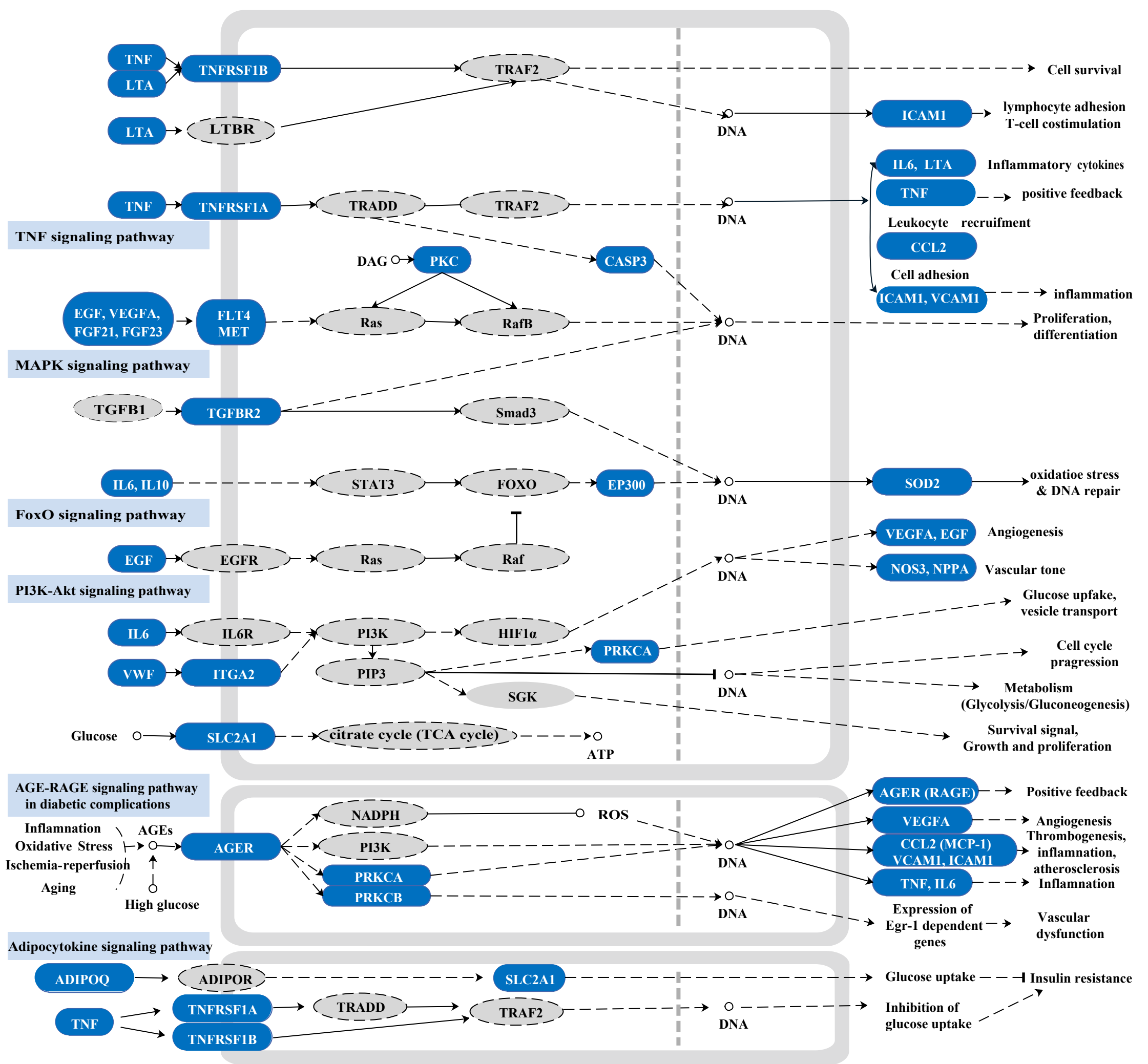

Supplement: Supplementary file 1 — Additional file 1: Table S1. Key words for search of prognosis literature on DN in Pubmed. Table S2. Key words for search of prognosis literature on IgAN, IMN, pFSGS and LN in Pubmed. Figure S1. The workflow for data processing. Figure S2. Prognostic markers in DN subtypes. Figure S3. Specimen sources of DN prognostic markers. Figure S4. Prognostic genes and proteins of DN (Risk genes and proteins for DN progression). Figure S5. GO enrichment analysis of DN prognostic genes and proteins corresponding to different end point events. Figure S6. The distribution of DN prognostic molecules in the enriched KEGG pathways. Figure S7. Role and regulatory relationship of DN prognostic molecules based on animal/cell culture models. Figure S8. High-risk genes for DN development and progression. Figure S9. Interactions between DN prognostic microRNAs and proteins, genes. [file 12967_2019_2016_MOESM1_ESM.zip › Additional file 1 Figure S6.pdf]

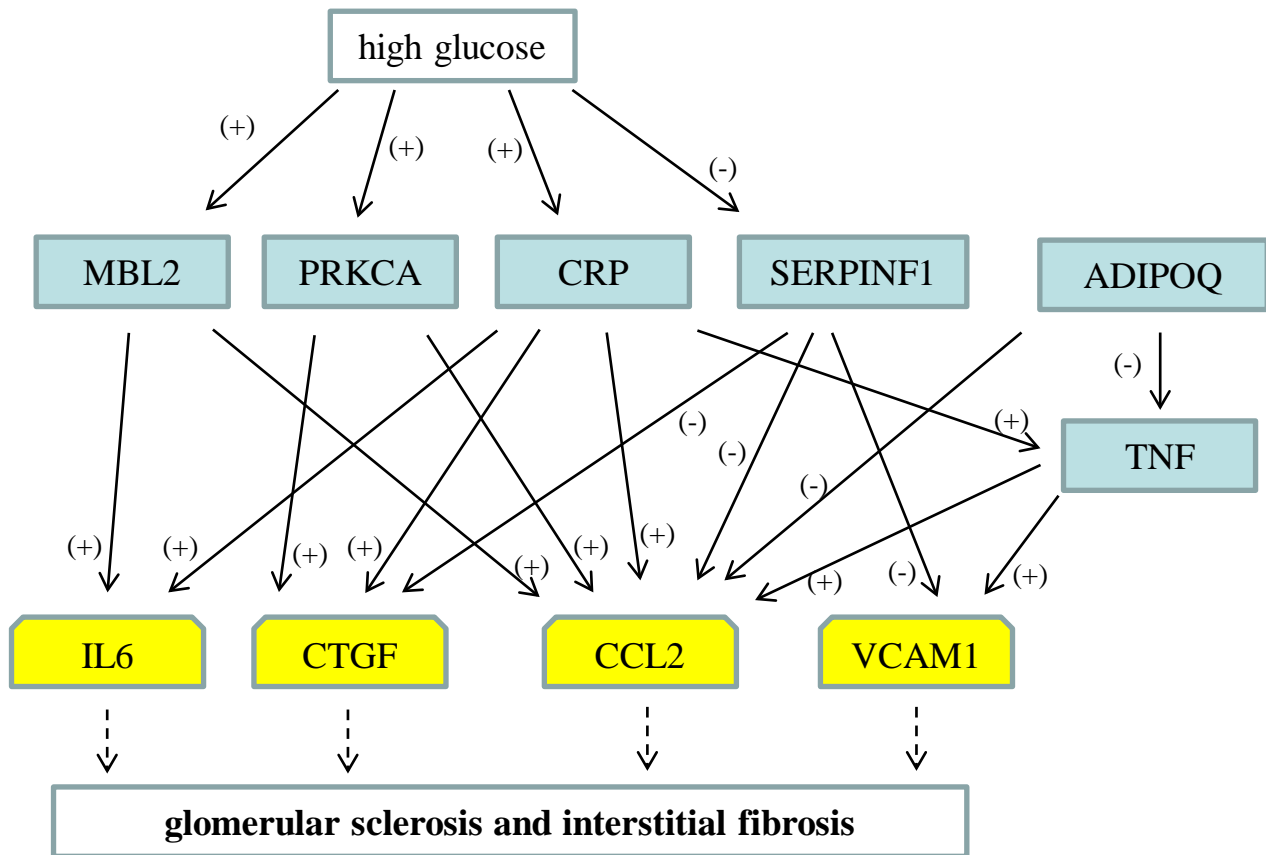

Supplement: Supplementary file 1 — Additional file 1: Table S1. Key words for search of prognosis literature on DN in Pubmed. Table S2. Key words for search of prognosis literature on IgAN, IMN, pFSGS and LN in Pubmed. Figure S1. The workflow for data processing. Figure S2. Prognostic markers in DN subtypes. Figure S3. Specimen sources of DN prognostic markers. Figure S4. Prognostic genes and proteins of DN (Risk genes and proteins for DN progression). Figure S5. GO enrichment analysis of DN prognostic genes and proteins corresponding to different end point events. Figure S6. The distribution of DN prognostic molecules in the enriched KEGG pathways. Figure S7. Role and regulatory relationship of DN prognostic molecules based on animal/cell culture models. Figure S8. High-risk genes for DN development and progression. Figure S9. Interactions between DN prognostic microRNAs and proteins, genes. [file 12967_2019_2016_MOESM1_ESM.zip › Additional file 1 Figure S7.pdf]

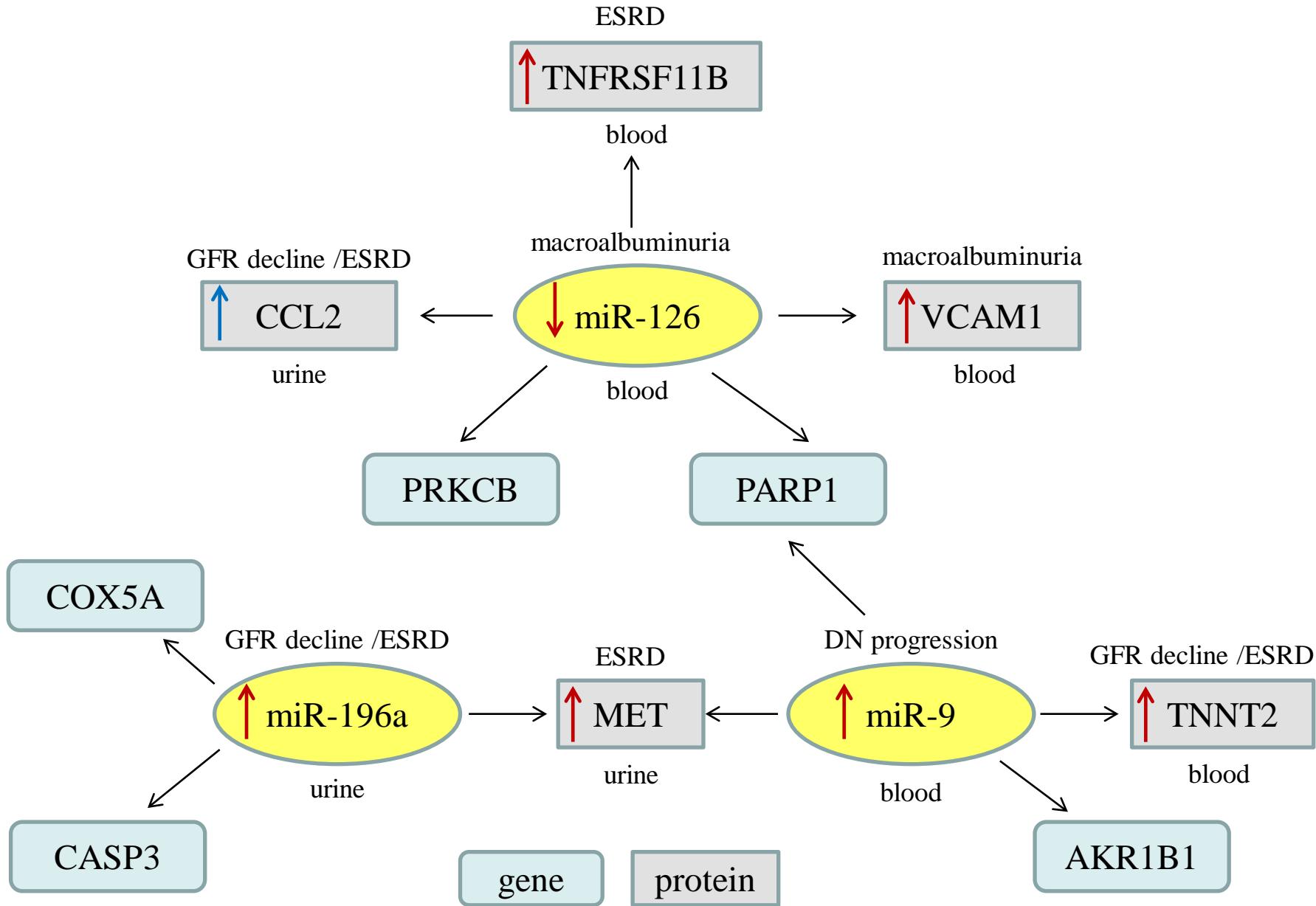

Supplement: Supplementary file 1 — Additional file 1: Table S1. Key words for search of prognosis literature on DN in Pubmed. Table S2. Key words for search of prognosis literature on IgAN, IMN, pFSGS and LN in Pubmed. Figure S1. The workflow for data processing. Figure S2. Prognostic markers in DN subtypes. Figure S3. Specimen sources of DN prognostic markers. Figure S4. Prognostic genes and proteins of DN (Risk genes and proteins for DN progression). Figure S5. GO enrichment analysis of DN prognostic genes and proteins corresponding to different end point events. Figure S6. The distribution of DN prognostic molecules in the enriched KEGG pathways. Figure S7. Role and regulatory relationship of DN prognostic molecules based on animal/cell culture models. Figure S8. High-risk genes for DN development and progression. Figure S9. Interactions between DN prognostic microRNAs and proteins, genes. [file 12967_2019_2016_MOESM1_ESM.zip › Additional file 1 Figure S9.pdf]

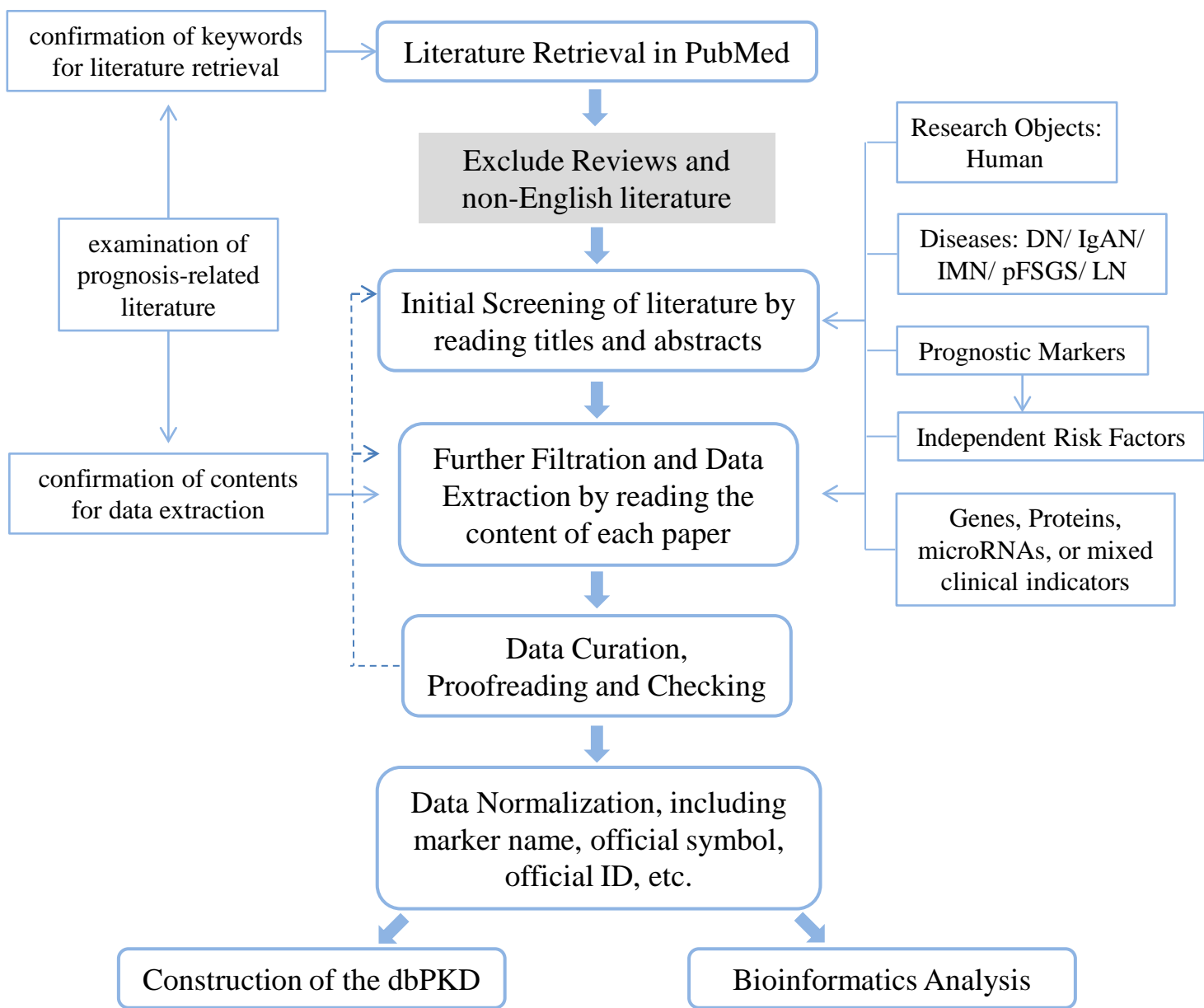

Supplement: Supplementary file 1 — Additional file 1: Table S1. Key words for search of prognosis literature on DN in Pubmed. Table S2. Key words for search of prognosis literature on IgAN, IMN, pFSGS and LN in Pubmed. Figure S1. The workflow for data processing. Figure S2. Prognostic markers in DN subtypes. Figure S3. Specimen sources of DN prognostic markers. Figure S4. Prognostic genes and proteins of DN (Risk genes and proteins for DN progression). Figure S5. GO enrichment analysis of DN prognostic genes and proteins corresponding to different end point events. Figure S6. The distribution of DN prognostic molecules in the enriched KEGG pathways. Figure S7. Role and regulatory relationship of DN prognostic molecules based on animal/cell culture models. Figure S8. High-risk genes for DN development and progression. Figure S9. Interactions between DN prognostic microRNAs and proteins, genes. [file 12967_2019_2016_MOESM1_ESM.zip › Additional file 1 Figure S1.pdf]

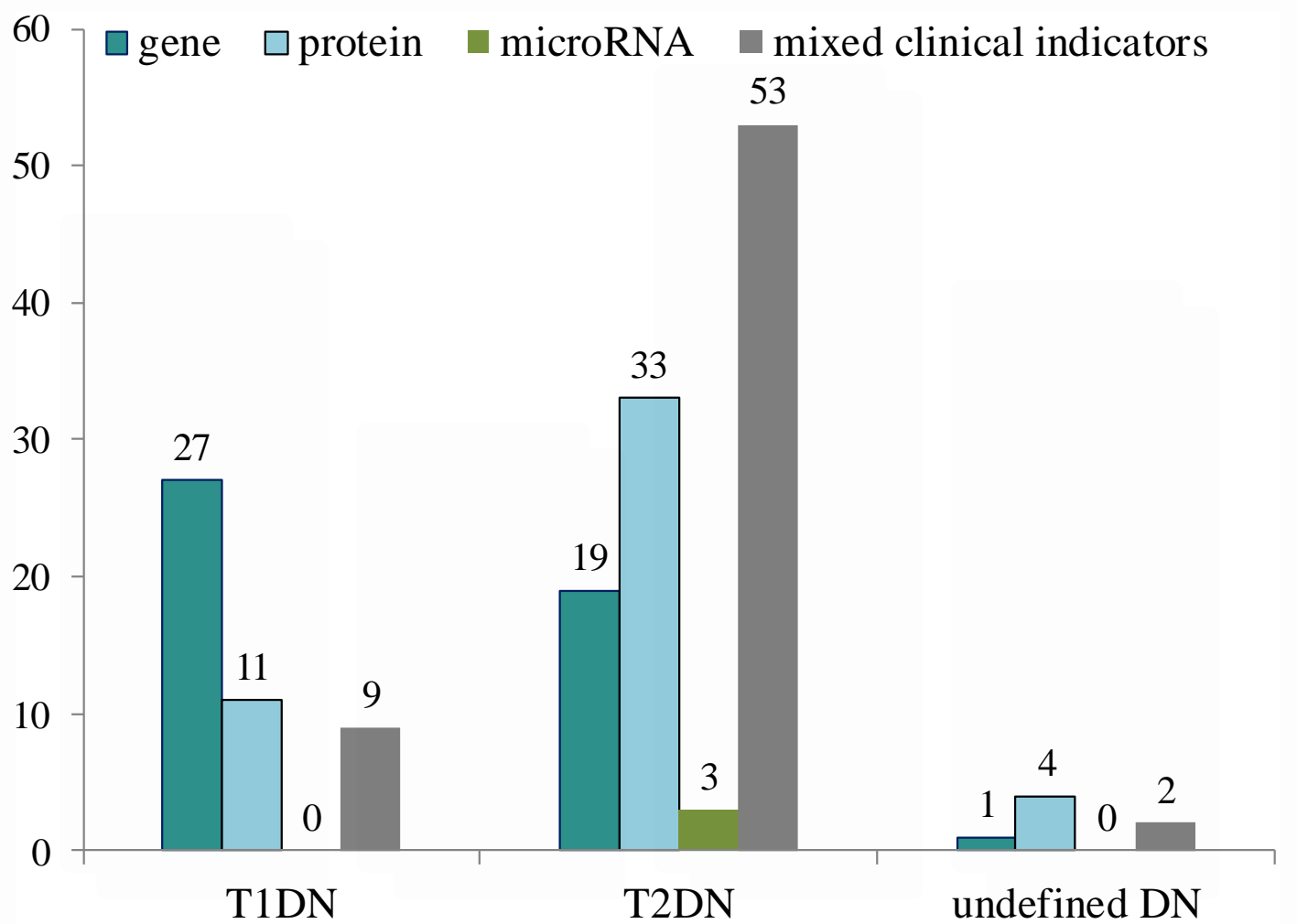

Supplement: Supplementary file 1 — Additional file 1: Table S1. Key words for search of prognosis literature on DN in Pubmed. Table S2. Key words for search of prognosis literature on IgAN, IMN, pFSGS and LN in Pubmed. Figure S1. The workflow for data processing. Figure S2. Prognostic markers in DN subtypes. Figure S3. Specimen sources of DN prognostic markers. Figure S4. Prognostic genes and proteins of DN (Risk genes and proteins for DN progression). Figure S5. GO enrichment analysis of DN prognostic genes and proteins corresponding to different end point events. Figure S6. The distribution of DN prognostic molecules in the enriched KEGG pathways. Figure S7. Role and regulatory relationship of DN prognostic molecules based on animal/cell culture models. Figure S8. High-risk genes for DN development and progression. Figure S9. Interactions between DN prognostic microRNAs and proteins, genes. [file 12967_2019_2016_MOESM1_ESM.zip › Additional file 1 Figure S2.pdf]

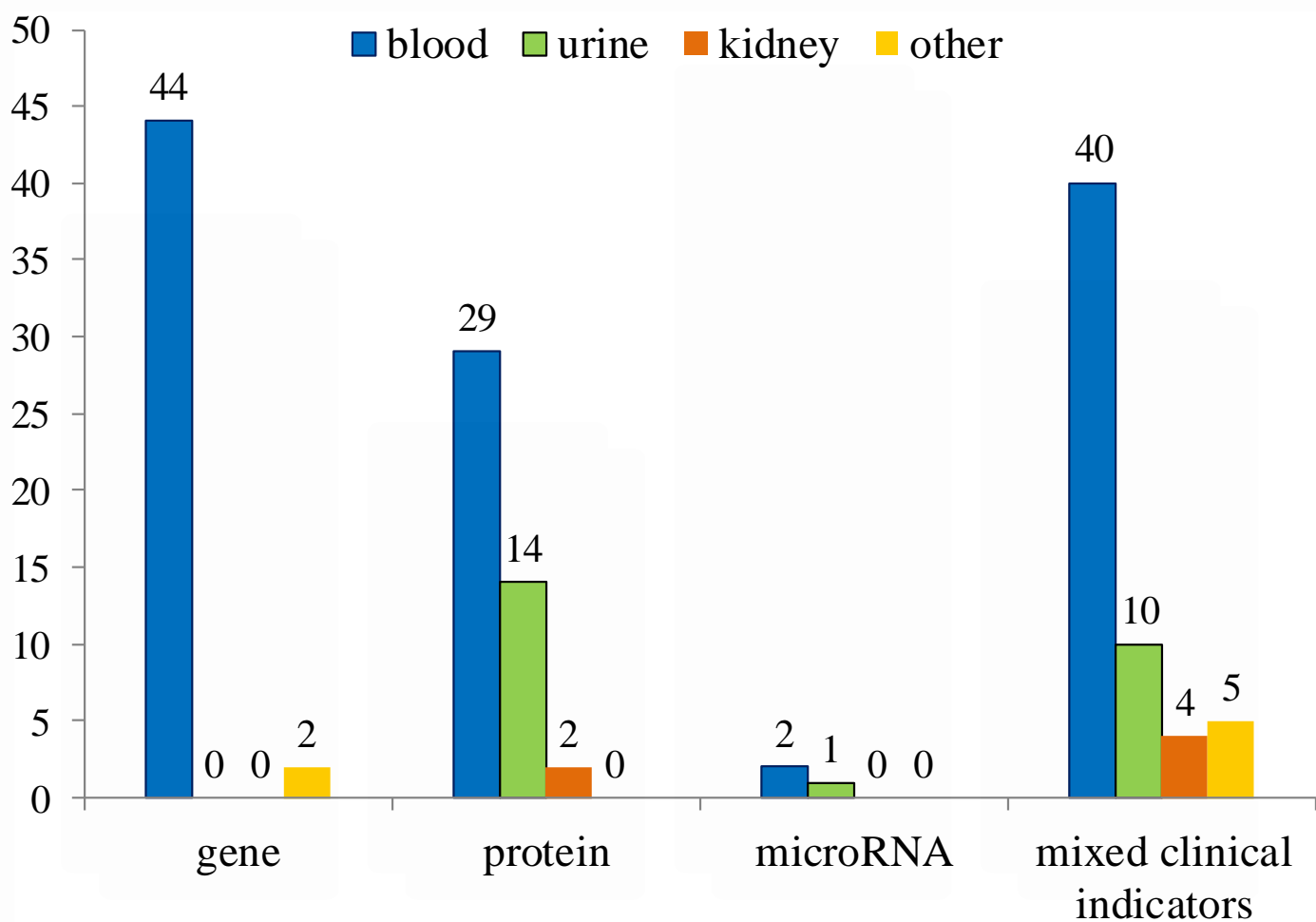

Supplement: Supplementary file 1 — Additional file 1: Table S1. Key words for search of prognosis literature on DN in Pubmed. Table S2. Key words for search of prognosis literature on IgAN, IMN, pFSGS and LN in Pubmed. Figure S1. The workflow for data processing. Figure S2. Prognostic markers in DN subtypes. Figure S3. Specimen sources of DN prognostic markers. Figure S4. Prognostic genes and proteins of DN (Risk genes and proteins for DN progression). Figure S5. GO enrichment analysis of DN prognostic genes and proteins corresponding to different end point events. Figure S6. The distribution of DN prognostic molecules in the enriched KEGG pathways. Figure S7. Role and regulatory relationship of DN prognostic molecules based on animal/cell culture models. Figure S8. High-risk genes for DN development and progression. Figure S9. Interactions between DN prognostic microRNAs and proteins, genes. [file 12967_2019_2016_MOESM1_ESM.zip › Additional file 1 Figure S3.pdf]
